# Association between work environment changes due to the COVID‐19 pandemic and post‐traumatic stress disorder in Japanese workers during the emergency declaration

**DOI:** 10.1002/pcn5.206

**Published:** 2024-06-14

**Authors:** Tetsuro Noda, Kumi Hirokawa, Kyoko Tokunaga

**Affiliations:** ^1^ Higashi Fuse Noda Clinic Higashiosaka Japan; ^2^ Osaka University of Human Science Settsu Japan; ^3^ Faculty of Societal Safety Sciences, Kansai University Takatsuki Japan; ^4^ Team HUMAN LLC Osaka Japan

**Keywords:** COVID‐19 pandemic, Japanese workers, mental health, online work, post‐traumatic stress disorder (PTSD)

## Abstract

**Aim:**

This study investigated the association between COVID‐19 pandemic‐related work environment changes and suspected post‐traumatic stress disorder (PTSD) in Japanese workers.

**Methods:**

A web survey of 1104 workers was conducted in Japan between February 24 and March 2, 2021. The Japanese version of the Impact of Event Scale–Revised and questions regarding work environments and COVID‐19 pandemic‐related lifestyle changes were used.

**Results:**

PTSD was suspected in 19.7% of respondents and was significantly higher in men (22.2%) than in women (17.2%). Being older and having an independent business were associated with decreased suspected PTSD risk. Longer online work hours, decreased sleep duration, and alcoholism were associated with increased suspected PTSD risk. When stratified by sex, long online work hours and fewer years of service were associated with increased suspected PTSD risk in men. An association between alcoholism and suspected PTSD was also observed in men. Younger age and decreased sleep duration were significantly associated with suspected PTSD in women.

**Conclusion:**

Younger men with shorter work service duration were particularly vulnerable to pandemic‐related PTSD, emphasizing the risks associated with long online work hours and alcoholism in men. Decreased sleep duration was a PTSD predictor in both sexes, suggesting its importance in PTSD prevention strategies for workers.

## INTRODUCTION

In Japan, the number of COVID‐19‐infected persons began to increase rapidly from mid‐March 2020, creating the first wave. The first state of emergency was declared on April 7, 2020. The state of emergency was lifted on May 25, 2020, as the disease outbreak began to be contained; however, workers were encouraged to continue working at home or go to their places of work at staggered times. Emergency and spread control‐related declarations were issued whenever there was an outbreak of COVID‐19.

Emergency declarations are government requests that citizens should refrain from leaving their homes, schools, or welfare facilities. These declarations did not have the force or penalty power of lockdowns, as was the case in other countries.

Isolation due to the COVID‐19 pandemic is believed to have caused various psychological reactions, including anxiety, depression, post‐traumatic stress disorder (PTSD), psychological stress, and stress.^1^, ^2^, ^3^

The incidence of PTSD has reportedly increased because of the COVID‐19 pandemic.^1^, ^2^, ^3^, ^4^, ^5^, ^6^, ^7^, ^8^, ^9^, ^10^ There is some debate as to whether the COVID‐19 pandemic can be considered a traumatic stressor that causes PTSD because the DSM‐5^11^ states that a life‐threatening or debilitating medical illness is not necessarily considered a traumatic event.^12^ However, extensive research has supported the idea that COVID‐19 can be understood as a traumatic stressor that elicits PTSD‐like reactions and exacerbates other related mental health problems.^13^, ^14^ Most studies on the relationship between the COVID‐19 pandemic and suspected PTSD have been conducted among high‐risk workers susceptible to the infection, such as healthcare workers. We could not find any literature on the general workforce, including that in Japan, therefore we investigated this association among the general Japanese workforce. This study focused on the actual suspected PTSD exhibited by workers due to the COVID‐19 pandemic and examined the factors that predict suspected PTSD in terms of workers’ demographic data, and changes in the work environment and lifestyles after the COVID‐19 pandemic.

## METHODS

### Participants and procedure

A web survey of workers was requested by the INTAGE Corporation between February 24 and March 2, 2021, that is, during the second emergency declaration. The survey was sent to 5725 workers, of whom 1104 completed it (valid response rate 19.3%). Of the participants, 554 (50.2%) were male and 550 were female, ranging in age from 20 to 69 years, with a mean of 47.3 (standard deviation [SD] = 13.6) years.

### Questionnaires

#### Suspected PTSD

The Japanese version of the Impact of Event Scale‐Revised (IES‐R) was used to assess suspected PTSD.^15^ The IES‐R is a self‐report measure based on the *Diagnostic and Statistical Manual of Mental Disorders, Fourth Edition* (DSM‐IV).^16^ This scale consists of 22 items of difficulty in total (eight items of intrusion, eight items of avoidance, and six items of hyperarousal that occur after involvement with any type of strong stress). This measure contains items that assess how difficult certain aspects were in the past week using five measures ranging from 0 (not at all) to 4 (extremely). Total scores can range from 0 to 88 points, and PTSD is suspected when individuals score ≥25 points. For the Great Hanshin‐Awaji Earthquake, the relevant findings were as follows: sensitivity = 0.75, specificity = 0.71, positive predictive value = 0.44, and negative predictive value = 0.90. The experience of stress was limited to the first state of emergency declaration in Japan. Cronbach's alpha for this instrument was 0.962.

#### Work‐related factors

We asked participants about their household income, employment status (i.e., regular employees, non‐regular employees, or independent businesses), length of employment duration, and daily online work hours. They were also asked whether there were any changes in their working hours due to the COVID‐19 pandemic (i.e., decreased, remained unchanged, or increased).

#### Demographic information and lifestyle changes due to COVID‐19

We asked for demographic information, such as sex, age, marital status, years of education, and whether the participants were currently receiving treatment for any mental illness. Lifestyle changes after the first emergency declaration included questions regarding sleep duration, weight, and alcohol intake (no habit, decreased, unchanged, or increased). In addition, to understand the participants' alcohol problems, we used one of the most popular alcohol screening questionnaires,^17^ the CAGE questionnaire, which focuses on cutting down, annoyance caused by criticism, guilt, and eye‐openers. Each question received a score of 1 for a “yes” response and 0 for “no.” A total score of 2 or greater was considered clinically significant. Cronbach's alpha for the questionnaire was 0.686.

### Statistical analysis

The relevant data were analyzed using SPSS version 28.0. Descriptive statistics were calculated for each variable. Data were expressed as mean (SD), median (interquartile range [IQR]), or number (percentage). A chi‐square test was used to examine the relationship between IES‐R (<25 or ≥25) and subjects' demographic characteristics or lifestyle changes after the COVID‐19 pandemic, including the relationship between CAGE (2< or ≥2) and changes in alcohol consumption (increased, decreased, unchanged) after the COVID‐19 pandemic. A residual analysis was conducted when a significant association was found based on a chi‐square test. For univariate analysis because the IES‐R scores were not normally distributed according to the Kolmogorov–Smirnov test, a nonparametric test (Mann–Whitney *U*‐test) was used to assess the relationship between the IES‐R score, CAGE score, and sex. To explore associations with suspected PTSD, binary logistic regression analysis was employed to adjust for the potential confounding factors of age, other demographic information, and lifestyle factors. Logistic regression analyses were conducted for the total sample and separately for male and female respondents. Odds ratios (ORs) and 95% confidence intervals (CIs) were also determined. Two dummy variables (IES‐R score of less than 25 = 0 and IES‐R score of 25 or more = 1) were used as dependent variables, and the independent variables were the work environment changes caused by the COVID‐19 pandemic after adjusting for potential confounding factors. Potential confounding factors were selected based on their associations with suspected PTSD. In the logistic regression analyses, all missing values were included in the model as dummy variables.

## RESULTS

### IES‐R score

A total of 1074 individuals participated in the IES‐R, whereas 30 did not respond. The median (IQR) IES‐R score was 8 (3–20). Although the median (IQR) IES‐R score was 8 (2–22) for men and 9 (3–19) for women, which was not significantly different (p = 0.601), 19.7% of the respondents had an IES‐R score of ≥25. This was significantly higher in men (22.2%) than in women (17.2%) (p = 0.031).

### Relationship between demographic characteristics and suspected PTSD

Table 1 shows the relationship between demographic characteristics and IES‐R scores (<25 or ≥25). No significant relationship was found between years of education or annual family income and the IES‐R score (<25 or ≥25). For marital status, there was no significant relationship between the IES‐R score (<25 or ≥25) in the total sample and male marital status. Among women, the proportion of unmarried persons with IES‐R ≥ 25 was significantly higher (p = 0.023). For employment status, there was no significant relationship between IES‐R score (<25 or ≥25) in both men and women. However, in the total sample, there were significantly more regular employees and fewer independent businesses having IES‐R ≥ 25 (p = 0.017). Years of service showed no significant relationship with IES‐R score (<25 or ≥25) in the total sample and among women, but men working for more than 5 years had a significantly lower IES‐R of ≥25 (p < 0.001). The relationship between current psychiatric treatment and IES‐R score (<25 or ≥25) formed a significantly higher percentage (IES‐R ≥ 25) in the total sample and among women with current treatment history (p = 0.017 and p = 0.029, respectively).

**Table 1:** Relationship between demographic characteristics and suspected PTSD.

		Total sample				Man				Woman			
Characteristics		Total	IES‐R < 25	IES‐R ≧ 25	Statistics	Total	IES‐R < 25	IES‐R ≧ 25	Statistics	Total	IES‐R < 25	IES‐R ≧ 25	Statistics
		1074 (100.0)	862 (100.0)	212 (100.0)		544 (100.0)	423 (100.0)	121 (100.0)		530 (100.0)	439 (100.0)	91 (100.0)	
Years of education	≦9	16 (1.5)	11 (1.3)	5 (2.4)	*χ* ^2^ = 4.96, df = 2	7 (1.3)	5 (1.2)	2 (1.7)	χ^2^ = 4.39, df = 2	9 (1.7)	6 (1.4)	3 (3.3)	χ^2^ = 2.06, df = 2
	10–12	277 (25.8)	212 (24.6)	65 (30.7)	p = 0.084	137 (25.2)	98 (23.2)	39 (32.0)	p = 0.111	140 (26.4)	114 (26.0)	26 (28.6)	p = 0.357
	≧13	781 (72.7)	639 (74.1)	142 (67.0)		400 (73.5)	320 (75.7)	80 (6.1)		381 (71.9)	319 (72.7)	62 (68.1)	
Marital status	Married	602 (56.1)	492 (57.1)	110 (51.9)	*χ* ^2^ = 1.86, df = 1	335 (61.6)	261 (61.7)	74 (61.2)	χ^2^ = 0.01, df = 1	267 (50.4)	231 (52.6)	36 (39.6)	χ^2^ = 5.142, df = 1
	Unmarried	472 (43.9)	370 (42.9)	102 (48.1)	p = 0.173	209 (38.4)	162 (38.3)	47 (38.8)	p = 0.913	263 (49.6)	208 (47.4)	55 (60.4)	p = 0.023
Employment status	Regular employee	574 (53,4)	446 (51.7)^b^	128 (60.4)^a^	*χ* ^2^ = 8.17, df = 2	394 (72.4)	299 (70.7)	95 (78.5)	χ^2^ = 5.32, df = 2	180 (34.0)	147 (33.5)	33 (36.3)	χ^2^ = 1.63, df = 2
	Non‐regular work	397 (37.0)	324 (37.6)	73 (34,4)	p = 0.017	87 (16.0)	68 (16.1)	19 (15.7)	p = 0.07	310 (58.5)	256 (58.3)	54 (59.3)	p = 0.442
	Independent business	103 (9.6)	92 (10.7)^a^	11 (5.2)^b^		63 (11.6)	56 (13.2)	7 (5.8)		40 (7.5)	36 (8.2)	4 (4.4)	
Years of service	<1	118 (11.0)	92 (10.7)	26 (12.3)	*χ* ^２^ = 5.31, df = 2	47 (8.6)	31 (7.3)^b^	16 (13.2)^a^	χ^2^ = 14.72, df = 2	71 (13.4)	61 (13.9）	10 (11.0)	χ^2^ = 0.60, df = 2
	1–4	305 (28.4)	233 (27.0)	72 (34.0)	p = 0.07	123 (22.6)	84 (19.9)^b^	39 (32.2)^a^	p < 0.001	182 (34.3)	149 (33.9)	33 (36.3)	p = 0.741
	≧5	651 (60.6)	537 (62.3)	114 (53.8)		374 (68.8)	308 (72.8)^a^	66 (54.5)^b^		277 (52.3)	229 (52.2)	48 (52.7)	
Annual family income(×10^4^ JPY)	<300	194 (18.1)	149 (17.3)	45 (21.2)	*χ* ^2^ = 3.66, df = 4	64 (11.8)	48 (11.3)	16 (13.2)	χ^2^ = 4.733, df = 4	130 (24.5)	101 (23.0)	29 (31.9)	χ^2^ = 3.46, df = 4
	300–499	252 (23.5)	204 (23.7)	48 (22.6)	p = 0.454	138 (25.4)	107 (25.3)	31 (25.6)	p = 0.316	114 (21.5)	97 (22.1)	17 (18.7)	p = 0.484
As of February 26, 2021, 1 yen was 0.009 USD	500–999	356 (33.1)	286 (33.2)	70 (33.0)		213 (39.2)	164 (38.8)	49 (40.5)		143 (27.0)	122 (27.8)	21 (23.1)	
	≧1000	100 (9.3)	86 (10.0)	14 (6.6)		70 (12.9)	61 (14.4)	9 (7.4)		30 (5.7)	25 (5.7)	5 (5.5)	
	Unknown	172 (16.0)	137 (15.9)	35 (16.5)		59 (10.8)	43 (10.2)	16 (13.2)		113 (21.3)	94 (21.4)	19 (20.9)	
Mental illness requiring treatment	Yes	1037 (96.6)	838 (97.2)	199 (93.9)	*χ* ^2^ = 5.73, df = 1	523 (96.1)	409 (96.7)	114 (94.2)	χ^2^ = 1.55, df = 1	514 (97.0)	429 (97.7)	85 (93.4)	χ^2^ = 4.80, df = 1
	No	37 (3.4)	24 (2.8)	13 (6.1)	p = 0.017	21 (3.9)	14 (3.3)	7 (5.8)	p = 0.213	16 (3.0)	10 (2.3)	6 (6.6)	p = 0.029
CAGE score	<2	961 (89.5)	785 (91.0)	176 (83.0)	*χ* ^2^ = 11.71, df = 1	465 (85.5)	374 (88.4)	91 (75.2)	χ^2^ = 13.23, df = 1	496 (93.6)	411 (93.6)	85 (93.4)	χ^2^ = 0.01, df = 1
	≧2	113 (10.5)	77 (8.9)	36 (17.0)	p < 0.001	79 (14.5)	49(11.6)	30(24.8)	p < 0.001	34 (6.4)	28 (6.4)	6 (6.6)	p = 0.939

*Note*: CAGE score ≥ 2 indicates suspected alcohol dependence. Data are shown as n (%).

^a^ Residual analysis that is significantly greater than expected.

^b^ Residual analysis that is significantly lesser than expected.

The median (IQR) CAGE score was 0 (0–1) for men and 0 (0–0) for women, with men scoring significantly higher (p < 0.001) than women. A CAGE score ≥2 represented 10.5% of the total, with 14.5% men and 6.4% women. In the total sample and among men, CAGE ≥ 2 accounted for a significantly higher IES‐R score of ≥25 (p < 0.001 for each).

### Relationship between lifestyle changes, work environment changes due to the COVID‐19 pandemic, and suspected PTSD

Table 2 shows the relationship between lifestyle changes, changes in the work environment after the state of emergency declaration, and suspected PTSD. Individuals with unchanged sleep hours had a significantly lower percentage of IES‐R ≥ 25, while those with decreased sleep hours had a higher percentage of IES‐R ≥ 25 in the total sample and among both men and women (p < 0.001 for all). As for weight gain, unchanged individuals had a low percentage of IES‐R ≥ 25 in the total sample. Both male participants who gained and lost weight had a high percentage of IES‐R > 25. Individuals with longer time spent working online during work hours had a higher percentage of IES‐R ≥ 25, while those with almost no time spent working online had a lower percentage of IES‐R ≥ 25. This trend was also observed in men.

**Table 2:** Relationship between lifestyle changes, changes in the work environment due to the COVID‐19 pandemic, and suspected PTSD.

		Total sample				Man				Woman			
Lifestyle changes		Total	IES‐R < 25	IES‐R ≧ 25		Total	IES‐R < 25	IES‐R ≧ 25		Total	IES‐R < 25	IES‐R ≧ 25	
		1074 (100.0)	862 (100.0)	212 (100.0)	Statistics	544 (100)	423 (100.0)	121 (100.0)	Statistics	530 (100.0)	439 (100.0)	91 (100.0)	Statistics
Sleep hours	Unchanged	826 (76.9)	689 (79.9)^a^	137 (64.6)^b^	*χ* ^2^ = 52.28, df = 2	431 (79.2)	352 (83.2)^a^	79 (65.3)^b^	χ^2^ = 27.92, df = 2	395 (74.5)	337 (76.8)^a^	58 (67.7)^b^	χ^2^ = 25.23, df = ２
	Increased	102 (9.5)	88 (10.2)	14 (6.6)	p < 0.001	39 (7.2)	31 (7.3)	8 (6.6)	p < 0.001	63 (11.9)	57 (13.0)	6 (6.6)	p ＜ 0.001
	Decreased	146 (13.6)	85 (9.9)^b^	61 (28.8)^a^		74 (13.6)	40 (9.5)^b^	34 (28.1)^a^		72 (13.6)	45 (10.3)^b^	27 (29.7)^a^	
Weight	Unchanged	681 (63.6)	561 (65.3)^a^	120 (56.6)^b^	*χ* ^2^ = 6.28, df = 2	366 (67.5)	302 (71.7)^a^	64 (52.9)^b^	χ^2^ = 15.57, df = 2	315 (59.5)	259 (59.1)	56 (61.5)	χ^2^ = 0.20, df = 2
	Increased	292 (27.3)	226 (26.3)	66 (31.1)	p = 0.043	125 (23.1)	86 (20.4)^b^	39 (32.2)^a^	p < 0.001	167 (31.6)	140 (32.0)	27 (29.7)	p = 0.905
	Decreased	98 (9.2)	72 (8.4)	26 (12.3)		51 (9.4)	33 (7.8)^b^	18 (14.9)^a^		47 (8.9)	39 (8.9)	8 (8.8)	
Working hours	Unchanged	775 (72.2)	625 (72.5)	150 (70.8)	*χ* ^2^ = 0.282, df = 2	409 (75.2)	321 (75.9)	88 (72.7)	χ^2^ = 0.504, df = 2	366 (69.1)	304 (69.2)	62 (68.1)	χ^2^ = 0.063, df = 2
	Increased	75 (7.0)	59 (6.8)	16 (7.5)	p = 0.868	37 (6.8)	28 (6.6)	9 (7.4)	p = 0.777	38 (7.2)	31 (7.1)	7 (7.7)	p = 0.969
	Decreased	224 (20.9)	178 (20.6)	46 (21.7)		98 (18.0)	74 (17.5)	24 (19.8)		126 (23.8)	104 (23.7)	22 (24.2)	
Time spent working online during work hours (excluding lunch and breaks)	Almost never (less than 20%)	799 (74.4)	653 (75.8)^a^	146 (68.9)^b^	χ² = 11.07, df = 3	382 (70.2)	302 (71.4)	80 (66.1)	χ² = 11.07, df = 3	417 (78.7)	351 (80.0)	66 (72.5)	χ² = 2.53, df = 3
	About half (40%–60%)	103 (9.6)	86 (10.0)	17 (8.0)	p = 0.011	65 (11.9)	56 (13.2)	9 (7.4)	p = 0.011	38 (7.2)	30 (6.8)	8 (8.8)	p = 0.47
	More than half (60%–80%)	43 (4.0)	33 (3.8)	10 (4.7)		25 (4.6)	19 (4.5)	6 (5.0)		18 (3.4)	14 (3.2)	4 (4.4)	
	Mostly (more than 80%)	129 (12.0)	90 (10.4)^b^	39 (18.4)^a^		72 (13.2)	46 (10.9)^b^	26 (21.5)^a^		57 (10.8)	44 (10.0)	13 (14.3)	
Alcohol intake	No habit	301 (28.0)	246 (28.5)	55 (25.9)	*χ* ^2^ = 2.07, df = 3	117 (21.5)	93 (22.0)	24 (19.8)	χ^2^ = 0.78, df = 3	184(34.7)	153(34.9)	31(34.1)	χ^2^ = 1.67, df = 3
	Increased	112 (10.4)	85 (9.9)	27 (12.7)	p = 0.559	65 (11.9)	48 (11.3)	17 (14.0)	p = 0.854	47 (8.9)	37 (8.4)	10 (11.0)	p = 0.644
	Unchanged	535 (49.8)	432 (50.1)	103 (48.6)		303 (55.7)	236 (55.8)	67 (55.4)		232 (43.8)	196 (44.6)	36 (39.6)	
	Decreased	126 (11.7)	99 (11,5)	27 (12.7)		59 (10.8)	46 (10.9)	13 (10.7)		67 (12.6)	53 (12.1)	14 (15.4)	

*Note*: Two males and one female missing in weight data. Missing values were excluded from the test. Data are shown as n (%).

^a^ Residual analysis that is significantly greater than expected.

^b^ Residual analysis that is significantly lesser than expected.

### Predictors of suspected PTSD

Table 3 shows the predictors of suspected PTSD. In the total sample, being female (OR = 0.69, 95% CI 0.47–0.99), being older (OR = 0.98, 95% CI 0.96–0.99), and having an independent business (OR = 0.46, 95% CI 0.23–0.94) were associated with reduced risks of IES‐R ≥ 25. Notably, participants with more time spent teleworking during work hours (OR = 1.99, 95% CI 1.26–3.14), decreased sleep hours (OR = 5.89, 95% CI 2.91–11.90), and CAGE ≥ 2 (OR = 2.00, 95% CI 1.25–3.19) were significantly associated with higher risks of IES‐R ≥ 25.

**Table 3:** Predictors of PTSD by logistic analysis.

		Total sample			Men			Women		
Parameter		OR	95% CI	p value	OR	95% Cl	p value	OR	95% CI	p value
Sex	Men		Reference							
	Women	0.69	0.47–0.99	0.046						
Age		0.98	0.96–0.99	<0.001	0.97	0.95–0.99	0.006	0.98	0.96–0.998	0.031
Marital status	Married		Reference			Reference			Reference	
	Unmarried	0.89	0.61–1.31	0.552	0.59	0.34–1.06	0.075	1.45	0.82–2.56	0.205
Mental illness requiring treatment	No		Reference			Reference			Reference	
	Yes	2.02	0.96–4.31	0.068	2.38	0.84–6.76	0.104	2.87	0.86–9.55	0.086
Years of education	≦9		Reference			Reference			Reference	
	10–12	0.47	0.15–1.49	0.201	0.85	0.14–5.03	0.856	0.32	0.07–1.50	0.146
	≧13	0.35	0.11–1.08	0.067	0.51	0.09–2.94	0.452	0.30	0.07–1.39	0.124
Annual family income (×10^4^ JPY)	<300		Reference			Reference			Reference	
As of February 26, 2021, 1 yen was 0.009 USD	300–499	0.75	0.43–1.32	0.316	0.96	0.40–2.32	0.921	0.72	0.36–1.46	0.363
	500–999	0.75	0.43–1.32	0.368	1.03	0.39–2.73	0.947	0.64	0.29–1.40	0.254
	≧1000	0.61	0.27–1.39	0.233	0.65	0.19–2.19	0.478	0.76	0.26–2.19	0.612
Employment status	Regular employee		Reference			Reference			Reference	
	Non‐regular work	0.87	0.57–1.34	0.533	0.80	0.38–1.72	0.57	1.07	0.61–1.88	0.817
	Independent business	0.46	0.23–0.94	0.034	0.49	0.19–1.23	0.127	0.51	0.15–1.72	0.278
Years of service	<1		Reference			Reference			Reference	
	1–4	1.08	0.62–1.88	0.777	0.95	0.43–2.10	0.899	1.44	0.63–3.28	0.392
	≧5	0.87	0.50–1.50	0.607	0.45	0.20–0.98	0.043	1.87	0.81–4.33	0.141
Time spent online working during work hours (excluding lunch and breaks)	Almost never (less than 20%)		Reference			Reference			Reference	
	About half (40%–60%)	0.93	0.51–1.68	0.798	0.69	0.30–1.59	0.38	1.53	0.64–3.71	0.342
	More than half (60%–80%)	1.62	0.74–3.53	0.224	1.72	0.59–4.98	0.319	1.64	0.47–5.75	0.443
	Mostly (more than 80%)	1.99	1.26–3.14	0.003	2.22	1.19–4.15	0.012	1.89	0.91–3.93	0.087
Weight	Unchanged		Reference			Reference			Reference	
	Increased	0.74	0.51–1.08	0.148	0.44	0.26–0.74	0.002	1.20	0.68–2.13	0.532
	Decreased	0.98	0.54–1.73	0.909	0.88	0.39–1.99	0.765	0.91	0.35–2.34	0.840
Sleep duration	Unchanged		Reference			Reference			Reference	
	Increased	1.58	0.83–3.00	0.164	1.61	0.60–4.33	0.343	2.07	0.81–5.34	0.131
	Decreased	5.89	2.91–11.90	<0.001	6.44	2.20–18.88	<0.001	7.09	2.53–19.86	<0.001
Working hours	Unchanged		Reference			Reference			Reference	
	Increased	1.65	0.86–3.17	0.132	2.49	0.98–6.35	0.056	1.14	0.44–2.98	0.785
	Decreased	1.56	0.76–3.19	0.227	2.23	0.78–6.36	0.132	1.35	0.48–3.83	0.571
CAGE score	<2		Reference			Reference			Reference	
	2<	2.00	1.25–3.19	0.004	2.54	1.43–4.51	0.001	0.94	0.35–2.56	0.910

*Note*: CAGE score ≥ 2 indicates suspected alcohol dependence.

Abbreviations: CI, confidence interval; OR, odds ratio.

For men, being older (OR = 0.97, 95% CI 0.95–0.99) and having years of service ≥5 (OR = 0.45, 95% CI 0.20–0.98) were associated with decreased risks of IES‐R ≥ 25. Men with more time spent teleworking during work hours (OR = 2.22, 95% CI 1.19–4.15), increased weight (OR = 0.44, 95% CI 0.26–0.74), decreased sleep hours (OR = 6.44, 95% CI 2.20–18.88), and CAGE ≥ 2 (OR = 2.54, 95% CI 1.43–4.51) were significantly associated with increased risks of IES‐R ≥ 25.

For women, only being younger (OR = 0.98, 95% CI 0.96–0.998) and having decreased sleep hours (OR = 7.09, 95% CI 2.53–19.86) were significantly associated with increased risks of IRS‐R ≥ 25.

## DISCUSSION

In the present study, the IES‐R was administered in response to the second state of emergency declaration in Japan, and 19.7% of the respondents showed suspected PTSD. A survey conducted by Noda et al.^10^ using the IES‐R as the first emergency declaration‐related stressor for undergraduate and graduate students in the Kansai area of Japan in July 2021 (after the withdrawal of the third state of emergency declaration) found that suspected PTSD was present in 13.4% of the participants. The present study demonstrated that the same tendency could be observed in Japanese workers. At the time of the first emergency declaration, COVID‐19 was an unknown virus; there was no vaccine for COVID‐19, and the mortality rates for people infected with COVID‐19 were high in China, Europe, and the United States, therefore the Japanese were fearful, and the emergency declaration itself may have been a stress factor that caused PTSD.

In the international literature, a meta‐analysis of primarily China‐based peer‐reviewed studies on COVID‐19‐related mental health issues published before May 12, 2020, reported that PTSD was found in 21.9% of both the general population and healthcare workers.^4^ A meta‐analysis of 76 articles investigating PTSD due to COVID‐19 conducted by Qiu et al.^5^ reported that 28.34% of the respondents displayed PTSD symptoms. In a study on the impact of the COVID‐19 pandemic in China, Iran, Malaysia, Pakistan, the Philippines, Thailand, and Vietnam, the so‐called seven middle‐income countries (MICs), the IES‐R average was the highest in Thailand at 42.4 and lowest in Vietnam at 17.4. Although not shown in the results of this study, we found an average IES‐R of 14.1, which was low compared with the relevant scores of other Asian countries.^18^ The incidence of PTSD among Japanese people tended to be lower than that in other countries, and the reasons for this need to be explored in the future.

For the total sample, in the multivariate analyses, the major influences on suspected PTSD were male sex and younger age. In general, youths and women are more likely to exhibit PTSD during the COVID‐19 pandemic.^8^ This study found that youths were more likely to exhibit PTSD symptoms; however, men were more likely to exhibit PTSD symptoms than women. A study of the general Japanese population (mean age = 52.76) conducted between 2020 and 2022 reported that men presented more PTSD symptoms than women.^19^ In our study, work environment, years of service, and time spent working online during work hours affected PTSD in men, whereas work environment did not affect PTSD in women. Men may be more prone to PTSD than women because they are more susceptible to the effects of the work environment in Japan.

Men with more than 5 years of work experience were significantly less likely to have PTSD, indicating that consideration should be given to male workers with less work experience. Approximately 70% of men and 80% of women reported that they rarely had online jobs, suggesting that even though the government encouraged workers to work at home to prevent infection, little progress had been made in this regard. A scoping study of 29 studies on teleworking and health before the COVID‐19 pandemic showed a decrease in subjective stress across six studies; the results for depression, anxiety, and other disorders varied across those studies.^20^ In particular, subjective stress in men decreased through online work, while subjective stress in women increased.^20^ However, the present study showed that long online work hours were associated with suspected PTSD in men. Psychological stress levels in online workers were lower than in commuters during the COVID‐19 pandemic, and when online workers received support from colleagues, their psychological stress levels were reduced.^21^ However, when online workers had a poor work environment at home, their psychological stress levels increased.^22^ Working from home for longer periods could lead to increased isolation, difficulty in maintaining a work–life balance, and disruption of daily routines, which could exacerbate the symptoms of PTSD. Associations between online work and mental health are generally inconsistent and unclear because of sex differences, family structures, and support from colleagues and supervisors.^22^ Intervention studies on online work involving factors such as sufficient support from supervisors and colleagues for workers with long online work hours are necessary.

Since the symptoms of PTSD include hyperarousal,^11^ it may be normal for participants with suspected PTSD to have decreased sleep duration after the COVID‐19 pandemic. However, there are reports that sleep quality worsened during the COVID‐19 pandemic^23^ and that the switch to remote work worsened sleep quality.^24^ With the COVID‐19 pandemic demanding stay‐at‐home restrictions, managing sleep will improve quality of life. Digital cognitive behavioral therapy for insomnia (CBT‐I) is not only effective^25^ but also cost‐effective^26^ and has been recommended during the COVID‐19 pandemic because of the difficulty in accessing medical care^27^ in Europe. In Japan, the practical application of CBT‐I has not yet progressed, therefore it will be necessary to promote it in the future.

Furthermore, especially for men, CAGE scores ≥2 implied that alcoholism was significantly associated with an increased risk of suspected PTSD. Increased alcohol consumption has been reported in other countries following the COVID‐19 pandemic.^28^, ^29^, ^30^, ^31^ Increased alcohol consumption has been reported to increase ORs for PTSD.^32^ In Japan, national alcohol consumption decreased markedly in the 2021 fiscal year.^33^ In our study, approximately 11% of men showed an increase or decrease in this regard, whereas 8.9% of women showed an increase, with 12.6% showing a decrease. However, for both men and women, the increase in alcohol consumption was significantly greater among those suspected of having alcoholism, according to the CAGE test, thus suggesting that alcohol consumption increased among those with preexisting alcohol problems, therefore attention should be paid to male workers with high alcohol consumption.

In the present study, only younger age and decreased sleep duration were associated with an increased risk of suspected PTSD among female workers, whereas work‐related factors were not significantly associated. The stronger association between work‐related factors and vulnerability to health problems among men compared with the corresponding association among women may be related to cultural and historical backgrounds. As traditional gender roles remain prevalent in Japanese society, many Japanese working women engage in non‐regular work, including temporary, contract, and part‐time jobs.^34^ Unmeasured factors such as family relationships and relationships with supervisors and colleagues should be considered. Further investigations of sex differences in the associations between work‐related factors and mental health among Japanese working women should be conducted.

## LIMITATIONS

The data used in this study had several limitations. As the survey was administered online, it may have been biased toward survey participants with computer skills. Furthermore, as self‐report screening tools were used instead of face‐to‐face evaluations, the occurrence frequency of suspected PTSD and alcoholism may have been overestimated. Additionally, because the questionnaires were self‐administered, the respondents' language abilities and level of comprehension regarding the questionnaire could have affected the results. A centralization tendency may have also occurred.

As PTSD is more prevalent in men than in women, susceptibility to the work environment and alcohol dependence were considered possible factors, whereas younger age and less sleep were identified as PTSD risk factors common to both sexes. However, it was not possible to establish a causal relationship between these life‐related factors and suspected PTSD due to the cross‐sectional study design. Despite these limitations, the data contributed to our knowledge of workers' mental health during the COVID‐19 pandemic.

## CONCLUSION

Approximately 20% of participants showed PTSD symptoms, suggesting that the COVID‐19 pandemic has worsened workers' mental health. In both men and women, decreased sleep duration was found to be a predictor of suspected PTSD that required attention. Longer hours of online work and fewer years of service were associated with an increased risk of suspected PTSD in men. In addition to these factors, the effects of factors such as alcoholism in men and younger age in both sexes should be considered. Although it is unclear whether these factors are the causes or consequences of suspected PTSD, they should be emphasized in mental health measures for Japanese workers.

## AUTHOR CONTRIBUTIONS

Tetsuro Noda: Conceptualization, data curation, formal analysis, project administration, writing—original draft, writing—review and editing. Kumi Hirokawa: Conceptualization, data curation, formal analysis, writing—review and editing. Kyoko Tokunaga: Conceptualization, data curation, and formal analysis.

## CONFLICT OF INTEREST STATEMENT

N/A.

## ETHICS APPROVAL STATEMENT

The ethics committee of the Hyogo University of Teacher Education approved this study (No. 2020−37).

## PATIENT CONSENT STATEMENT

All the participants were informed of the purpose of the study and that their participation was anonymous and voluntary. Written informed consent was obtained from all the participants.

## CLINICAL TRIAL REGISTRATION

N/A.

## Data Availability

The data that support the findings of this study are available from the corresponding author upon reasonable request.
